# Supplementary material for: The Small RNA Universe of Capitella teleta
Source: Front Mol Biosci. 2022 Feb 25;9:802814. doi: 10.3389/fmolb.2022.802814 (PMC8915122; doi:10.3389/fmolb.2022.802814)
Supplement: Supplementary file 1 [file DataSheet1.ZIP › Supplement/known/CAPTEscaffold_522_23007.pdf]

Provisional ID	: CAPTEscaffold_522_23007
Score total	: 268602.5
Score for star read(s)	: 3.9
Score for read counts	: 268592.8
Score for mfe	: 1.2
Score for randfold	: 1.6
Score for cons. seed	: 3
Total read count	: 526845
Mature read count	: 526840
Loop read count	: 0
Star read count	: 5

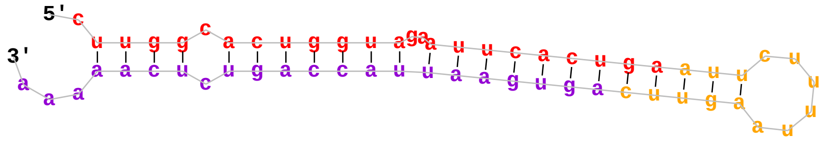

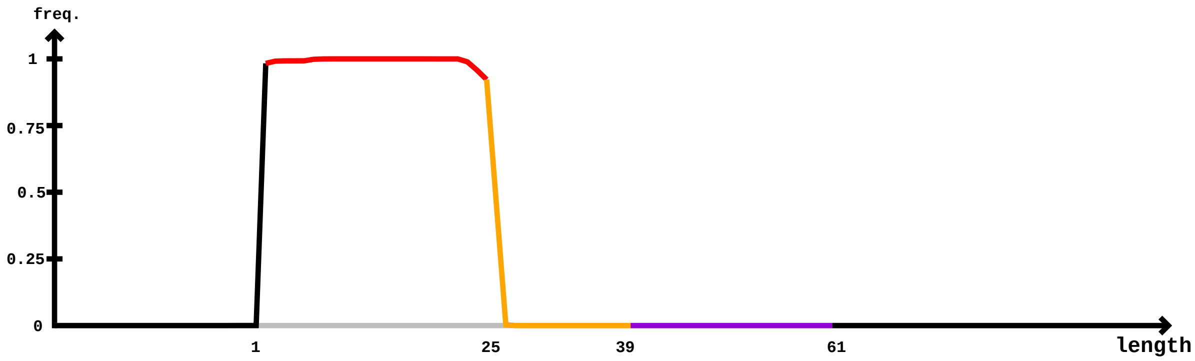

Mature Star

```
5'- uaaaggcgcucccaacccuucuuggcacugguagaauucacugaauucuuuuaaguucagugaauuaccagucucaaaaauugugagugaaccauugcggcguacuuuacggac   -3'   obs
    uaaaggcgcucccaacccuucuuggcacugguagaauucacugaauucuuuuaaguucagugaauuaccagucucaaaaauugugagugaaccauugcggcguacuuuacggac         exp
    uaaaggcgcucccaacccuucuuggcacugguagaauucacugaauucuuuuaaguucagugaauuaccagucucaaaaauugugagugaaccauugcggcguacuuuacggac         known
    ....(((((((.(((...((.((((.(((((((..((((((((((((......))))))))))))))))))).)))).))))).)))))..)).......((((...))))...    reads   mm   sample
    .................cuucuuggcacugguagaauu............................................................................    1       0    seq
    .................cuucuuggcacugguagaauucac.........................................................................    2       0    seq
    .................cuucuuggcacugguagaauucacu........................................................................    26      0    seq
    .................cuucuuggcacugguagaauucacugU......................................................................    1       1    seq
    .................cuucuuggcacugguagaauucacuAaa.....................................................................    1       1    seq
    ...................ucuuggcacugguagaauucacu........................................................................    2       0    seq
    ...................ucuuggcUcugguagaauucacug.......................................................................    1       1    seq
    ...................ucuuggcacugguagaauucacug.......................................................................    2       0    seq
    ...................ucuuggcacugguagaauucacugaa.....................................................................    1       0    seq
    ....................cuuggcacuggCagaauu............................................................................    1       1    seq
    ....................cuuAgcacugguagaauu............................................................................    1       1    seq
    ....................cuuggcacugguagaauu............................................................................    18      0    seq
    ....................cuuggcacugguagaauuc...........................................................................    7       0    seq
    ....................cuuggcUcugguagaauuca..........................................................................    1       1    seq
    ....................cuuggcacugguagaauuca..........................................................................    10      0    seq
    ....................cuNggcacugguagaauucac.........................................................................    1       1    seq
    ....................Auuggcacugguagaauucac.........................................................................    1       1    seq
    ....................cuuggcacugguagaauuAac.........................................................................    2       1    seq
    ....................cuuggcacGguagaauucac.........................................................................    2       1    seq
    ....................cuuggcacugguagaauGcac.........................................................................    1       1    seq
    ....................cuuggcacugguagUauucac.........................................................................    1       1    seq
    ....................cuuggcacugguUgaauucac.........................................................................    1       1    seq
    ....................cuuggcacuggCagaauucac.........................................................................    3       1    seq
    ....................cuuggcacugguaUaauucac.........................................................................    1       1    seq
    ....................Nuuggcacugguagaauucac.........................................................................    1       1    seq
    ....................cuuggcacuggGagaauucac.........................................................................    1       1    seq
    ....................cuuggcacugguagaauucac.........................................................................    5694    0    seq
    ....................cuuggcacuCguagaauucac.........................................................................    1       1    seq
    ....................cuuggcacugguagaUuucac.........................................................................    2       1    seq
    ....................cuuggcacugguagGauucac.........................................................................    2       1    seq
    ....................cuuggcacugUuagaauucac.........................................................................    1       1    seq
    ....................cuuggcacAgguagaauucac.........................................................................    1       1    seq
```

```
                    Mature                                Star
uaaaggcgcucccaacccuucuuggcacugguagaauucacugaauucuuuuaaguucagugaauuaccagucucaaaaauugugagugaaccauugcggcguacuuuacggac
....................cuuggcacugguagaaAucac.........................................................................  2      1   seq
....................cuugUcacugguagaauucac.........................................................................  2      1   seq
....................cuuggcGcugguagaauucac.........................................................................  1      1   seq
....................cCuggcacugguagaauucac.........................................................................  1      1   seq
....................cuuggcacugguagaauucGc.........................................................................  3      1   seq
....................cuuggcacuggAagaauucac.........................................................................  3      1   seq
....................cAuggcacugguagaauucac.........................................................................  4      1   seq
....................cuuCgcacugguagaauucac.........................................................................  2      1   seq
....................cuuggcacugguaAaauucac.........................................................................  1      1   seq
....................cuuAgcacugguagaauucac.........................................................................  6      1   seq
....................cuuggcacugguagaauuGac.........................................................................  1      1   seq
....................cuuggcacugguGgaauucac.........................................................................  3      1   seq
....................cuuggcacuUguagaauucac.........................................................................  2      1   seq
....................cuuggcacugguagaauCcac.........................................................................  1      1   seq
....................cuuggcacugguagaauAcac.........................................................................  2      1   seq
....................cuuggAacugguagaauucac.........................................................................  1      1   seq
....................cuuggcUcugguagaauucac.........................................................................  1      1   seq
....................cuAggcacugguagaauucac.........................................................................  2      1   seq
....................cuuggcacugguagaauuUac.........................................................................  3      1   seq
....................Uuuggcacugguagaauucac.........................................................................  4      1   seq
....................cuuggUacugguagaauucac.........................................................................  5      1   seq
....................cuuggcacuAguagaauucac.........................................................................  4      1   seq
....................Guuggcacugguagaauucac.........................................................................  2      1   seq
....................cuuggcacugguagUauucacu........................................................................  5      1   seq
....................cuuggcacugguUgaauucacu........................................................................  2      1   seq
....................Guuggcacugguagaauucacu........................................................................  1      1   seq
....................cuuggcacuggAagaauucacu........................................................................  4      1   seq
....................cuuggcacugguagaaGucacu........................................................................  3      1   seq
....................cuuggcacugguagaUuucacu........................................................................  4      1   seq
....................cuuggcacuAguagaauucacu........................................................................  15     1   seq
....................cuuggcacugguagaaCucacu........................................................................  5      1   seq
....................cuuggcacugguagaauucacN........................................................................  1      1   seq
....................cuuggUacugguagaauucacu........................................................................  6      1   seq
....................cuuggcacugguagaauuUacu........................................................................  26     1   seq
....................cuuggcacCgguagaauucacu........................................................................  1      1   seq
....................cuuAgcacugguagaauucacu........................................................................  12     1   seq
....................cuuggcacugguagaauCcacu........................................................................  1      1   seq
....................cuuggcGcugguagaauucacu........................................................................  7      1   seq
....................cuuggcacAgguagaauucacu........................................................................  3      1   seq
....................cuuggcacuCguagaauucacu........................................................................  3      1   seq
....................cuuggcCcugguagaauucacu........................................................................  2      1   seq
....................Nuuggcacugguagaauucacu........................................................................  5      1   seq
....................cuAggcacugguagaauucacu........................................................................  11     1   seq
....................cuuggcacugguagaGuucacu........................................................................  8      1   seq
....................cuuggAacugguagaauucacu........................................................................  1      1   seq
....................cuuggGacugguagaauucacu........................................................................  3      1   seq
....................cuuggcacugguagaauucGcu........................................................................  9      1   seq
....................cuuggcacugguagaauucacu........................................................................  16208  0   seq
....................cuuggcaAugguagaauucacu........................................................................  3      1   seq
....................cuuggcacugguagaaAucacu........................................................................  3      1   seq
....................cuuCgcacugguagaauucacu........................................................................  2      1   seq
....................cuuggcacugguagaauucacG........................................................................  6      1   seq
....................cuuggcacugguagaauucacC........................................................................  2      1   seq
....................cAuggcacugguagaauucacu........................................................................  8      1   seq
....................cCuggcacugguagaauucacu........................................................................  2      1   seq
....................cuugUcacugguagaauucacu........................................................................  2      1   seq
....................cuuggcacugguaAaauucacu........................................................................  4      1   seq
....................Uuuggcacugguagaauucacu........................................................................  8      1   seq
....................cuuggcacugCuagaauucacu........................................................................  1      1   seq
....................cuuggcacugAuagaauucacu........................................................................  4      1   seq
....................cuuggcacugguagGauucacu........................................................................  1      1   seq
....................cuuggcacuggCagaauucacu........................................................................  3      1   seq
....................cuuggcacugguagaauAcacu........................................................................  7      1   seq
....................cuuUgcacugguagaauucacu........................................................................  3      1   seq
....................cuuggcUcugguagaauucacu........................................................................  3      1   seq
....................cuuggcacugguagaauuGacu........................................................................  4      1   seq
....................cuuggcacugguGgaauucacu........................................................................  2      1   seq
....................cuugCcacugguagaauucacu........................................................................  3      1   seq
....................cuGggcacugguagaauucacu........................................................................  1      1   seq
....................cuuggcaUugguagaauucacu........................................................................  6      1   seq
```

```
                   Mature                  Star
uaaaggcgcucccaacccuucuuggcacugguagaauucacugaauucuuuuaaguucagugaauuaccagucucaaaaauugugagugaaccauugcggcguacuuuacggac
....................Auuggcacugguagaauucacu........................................................................ 3      1     seq
....................cuuggcacugguagaauucaAu........................................................................ 2      1     seq
....................cuCggcacugguagaauucacu........................................................................ 4      1     seq
....................cuuggcacugUuagaauucacu........................................................................ 1      1     seq
....................cNuggcacugguagaauucacu........................................................................ 1      1     seq
....................cuuggcacugguagaauucaGu........................................................................ 1      1     seq
....................cuuggcacugguagaauucacA........................................................................ 3      1     seq
....................cuuggcacugguagaauuAacu........................................................................ 3      1     seq
....................cuuggcacugguUgaauucacug....................................................................... 1      1     seq
....................cuuggcacugguagaauucacuA....................................................................... 38     1     seq
....................cuuggcacuUguagaauucacug....................................................................... 1      1     seq
....................cuugUcacugguagaauucacug....................................................................... 1      1     seq
....................cuuggcacGgguagaauucacug....................................................................... 1      1     seq
....................cuugAcacugguagaauucacug....................................................................... 4      1     seq
....................cCuggcacugguagaauucacug....................................................................... 2      1     seq
....................cuuggcaGugguagaauucacug....................................................................... 1      1     seq
....................cuuggcacugguagaauucGcug....................................................................... 3      1     seq
....................cuuggcacugguagaGuucacug....................................................................... 6      1     seq
....................cuCggcacugguagaauucacug....................................................................... 3      1     seq
....................Uuuggcacugguagaauucacug....................................................................... 11     1     seq
....................cuuggcacCgguagaauucacug....................................................................... 3      1     seq
....................Auuggcacugguagaauucacug....................................................................... 3      1     seq
....................cuuggcGcugguagaauucacug....................................................................... 9      1     seq
....................Nuuggcacugguagaauucacug....................................................................... 7      1     seq
....................cuuggcacugguagaauCcacug....................................................................... 6      1     seq
....................cuuggcacugguagaauucaAug....................................................................... 6      1     seq
....................cuuggcacugguagaauucacuN....................................................................... 3      1     seq
....................cuuggcacuggCagaauucacug....................................................................... 2      1     seq
....................cuuggcacugguGgaauucacug....................................................................... 6      1     seq
....................cuuggcacuCguagaauucacug....................................................................... 2      1     seq
....................cuuggcacugguagaauAcacug....................................................................... 5      1     seq
....................cuuggcacugguagUauucacug....................................................................... 3      1     seq
....................cuuggcacugguagaUuucacug....................................................................... 3      1     seq
....................cuuggcacugAuagaauucacug....................................................................... 1      1     seq
....................cuuUgcacugguagaauucacug....................................................................... 2      1     seq
....................cuuAgcacugguagaauucacug....................................................................... 16     1     seq
....................cuuggcacugguagaauuUacug....................................................................... 8      1     seq
....................cuuggcacugguagaauucacGg....................................................................... 14     1     seq
....................Guuggcacugguagaauucacug....................................................................... 1      1     seq
....................cuuggGacugguagaauucacug....................................................................... 2      1     seq
....................cuuggcacugguagaauucacug....................................................................... 17851  0     seq
....................cuuggcacugguagaauuAacug....................................................................... 8      1     seq
....................cuuggcacugguagaauucacCg....................................................................... 2      1     seq
....................cuuggcacAgguagaauucacug....................................................................... 6      1     seq
....................cuuggUacugguagaauucacug....................................................................... 12     1     seq
....................cuuggcUcugguagaauucacug....................................................................... 4      1     seq
....................cuuggcacugguagGauucacug....................................................................... 8      1     seq
....................cuuggcacugguagaauucCcug....................................................................... 1      1     seq
....................cuuggcacugguagaauucaGug....................................................................... 3      1     seq
....................cuuggcacugUuagaauucacug....................................................................... 2      1     seq
....................cuuggcacugguagaauGcacug....................................................................... 1      1     seq
....................cuuggcacugguagaauuGacug....................................................................... 2      1     seq
....................cNuggcacugguagaauucacug....................................................................... 1      1     seq
....................cuuggcacuAguagaauucacug....................................................................... 15     1     seq
....................cuuggcacugguagaauucUcug....................................................................... 1      1     seq
....................cuuggcacugguagaauucacuU....................................................................... 79     1     seq
....................cuuggcacugguaAaauucacug....................................................................... 4      1     seq
....................cuuggcacugguagaauucacAg....................................................................... 6      1     seq
....................cuuggcacugguagaaCucacug....................................................................... 2      1     seq
....................cuAggcacugguagaauucacug....................................................................... 14     1     seq
....................cuuggAacugguagaauucacug....................................................................... 10     1     seq
....................cuuggcacugguaUaauucacug....................................................................... 3      1     seq
....................cuuggcacugguagaaAucacug....................................................................... 10     1     seq
....................cuuggcaAugguagaauucacug....................................................................... 3      1     seq
....................cuuggcacuggAagaauucacug....................................................................... 6      1     seq
....................cAuggcacugguagaauucacug....................................................................... 4      1     seq
....................cuugCcacugguagaauucacug....................................................................... 2      1     seq
....................cuuggcCcugguagaauucacug....................................................................... 3      1     seq
....................cuuggcacugCuagaauucacug....................................................................... 1      1     seq
....................cuGggcacugguagaauucacug....................................................................... 2      1     seq
```

Mature **Star**

```
uaaaggcgcucccaacccuucuuggcacugguagaauucacugaauucuuuuaaguucagugaauuaccagucucaaaaauugugagugaaccauugcggcguacuuuacggac
....................cuuggcacugguagaauucacuC.......................................................................   1       1   seq
....................cuuggcacugguagaauCcacuga......................................................................   50      1   seq
....................cuuggcacugguaCaauucacuga......................................................................   29      1   seq
....................cuuggcCcugguagaauucacuga......................................................................   33      1   seq
....................cuuggcacugguagaauGcacuga......................................................................   46      1   seq
....................cuuggcacugguagaCuucacuga......................................................................   3       1   seq
....................cuuggcacugguagaaAucacuga......................................................................   188     1   seq
....................cuuggcacugguUgaauucacuga......................................................................   61      1   seq
....................cuuggcacugguagaauucGcuga......................................................................   142     1   seq
....................cuuggcacugguagaauucacuAa......................................................................   323     1   seq
....................cuugCcacugguagaauucacuga......................................................................   37      1   seq
....................cuuggcacugguagaauucacugN......................................................................   1       1   seq
....................cuuggcacuAguagaauucacuga......................................................................   377     1   seq
....................Guuggcacugguagaauucacuga......................................................................   47      1   seq
....................cuuggcacugguagaauucaGuga......................................................................   43      1   seq
....................cuuggcacugguagaauuAacuga......................................................................   168     1   seq
....................cuuggcacugguagaauuGacuga......................................................................   73      1   seq
....................cuuggcacuCguagaauucacuga......................................................................   51      1   seq
....................cuuggcacuUguagaauucacuga......................................................................   63      1   seq
....................cGuggcacugguagaauucacuga......................................................................   10      1   seq
....................cuuggcacugguaAaauucacuga......................................................................   84      1   seq
....................cuCggcacugguagaauucacuga......................................................................   93      1   seq
....................cuuggcacugguagGauucacuga......................................................................   99      1   seq
....................cuuggcacugUuagaauucacuga......................................................................   48      1   seq
....................Nuuggcacugguagaauucacuga......................................................................   123     1   seq
....................cuuggcacugguagaauucacuga......................................................................   454317  0   seq
....................cuuggcacugCuagaauucacuga......................................................................   27      1   seq
....................cuuggcacugguagaauucacugG......................................................................   1162    1   seq
....................cuuggcacugguagaGuucacuga......................................................................   162     1   seq
....................cuuggcacugguagaaCucacuga......................................................................   68      1   seq
....................cuuggcacuggAagaauucacuga......................................................................   161     1   seq
....................cuuggcacugguaUaauucacuga......................................................................   43      1   seq
....................cuuggcacugguagaauucaNuga......................................................................   4       1   seq
....................cuuggcacugguagaauucaAuga......................................................................   50      1   seq
....................cuuggcacugguagaUuucacuga......................................................................   125     1   seq
....................cuuggcaUugguagaauucacuga......................................................................   64      1   seq
....................cuugAcacugguagaauucacuga......................................................................   99      1   seq
....................cuuggcacuNguagaauucacuga......................................................................   3       1   seq
....................cuuCgcacugguagaauucacuga......................................................................   44      1   seq
....................cuuggcacugguagaauucacGga......................................................................   57      1   seq
....................cuuggcacugguagaauucacugC......................................................................   20      1   seq
....................cAuggcacugguagaauucacuga......................................................................   215     1   seq
....................Uuuggcacugguagaauucacuga......................................................................   349     1   seq
....................cuuggcacugguagaauAcacuga......................................................................   145     1   seq
....................cuuggcacGgguagaauucacuga......................................................................   41      1   seq
....................cuAggcacugguagaauucacuga......................................................................   364     1   seq
....................cuuggcacCgguagaauucacuga......................................................................   49      1   seq
....................cuuggcacugguagCauucacuga......................................................................   3       1   seq
....................cuuggcaGugguagaauucacuga......................................................................   26      1   seq
....................cuuggcacugguagaauucacuNa......................................................................   7       1   seq
....................cuuggcacugguagaauuUacuga......................................................................   332     1   seq
....................cuuggcacugguagaauucacugU......................................................................   1169    1   seq
....................cuuggcaAugguagaauucacuga......................................................................   63      1   seq
....................cuuUgcacugguagaauucacuga......................................................................   51      1   seq
....................cuuggcGcugguagaauucacuga......................................................................   197     1   seq
....................cuuggcacugguagaaGucacuga......................................................................   72      1   seq
....................cuuggcacugguCgaauucacuga......................................................................   8       1   seq
....................cuuggAacugguagaauucacuga......................................................................   179     1   seq
....................cuuggGacugguagaauucacuga......................................................................   92      1   seq
....................cuuggcacugguagaauucUcuga......................................................................   91      1   seq
....................cuuggcacugguagaaNucacuga......................................................................   1       1   seq
....................cuuggcacugguagaauucaUuga......................................................................   35      1   seq
....................cuuggNacugguagaauucacuga......................................................................   8       1   seq
....................cuuggcacugguagaauucNcuga......................................................................   1       1   seq
....................cuuggUacugguagaauucacuga......................................................................   190     1   seq
....................cuuggcacugguagaauucacuCa......................................................................   27      1   seq
....................cuugUcacugguagaauucacuga......................................................................   54      1   seq
....................cuuggcacugguagUauucacuga......................................................................   63      1   seq
....................cuuggcUcugguagaauucacuga......................................................................   79      1   seq
....................cuuggcacuggCagaauucacuga......................................................................   87      1   seq
```

Mature **Star**

uaaaggcgcucccaacccuucuuggcacugguagaauucacugaauucuuuuaaguucagugaauuaccagucucaaaaauugugagugaaccauugcggcguacuuuacggac

....................cuuggcacugguagaNuucacuga..	1	1	seq
....................cuuggcacugguagaauucacCga..	71	1	seq
....................cuuggcacugguagaauucacAga..	151	1	seq
....................cuuggcacAgguagaauucacuga..	72	1	seq
....................cuuggcacugguagaauucCcuga..	23	1	seq
....................cNuggcacugguagaauucacuga..	20	1	seq
....................cuuAgcacugguagaauucacuga..	300	1	seq
....................cuGggcacugguagaauucacuga..	29	1	seq
....................cuuggcacuggGagaauucacuga..	13	1	seq
....................cCuggcacugguagaauucacuga..	40	1	seq
....................cuuggcacugguagaauucacuUa..	40	1	seq
....................Auuggcacugguagaauucacuga..	56	1	seq
....................cuuggcacugguaNaauucacuga..	1	1	seq
....................cuuggcacugAuagaauucacuga..	43	1	seq
....................cuuggcacugguGgaauucacuga..	136	1	seq
....................cuuggcacugguagaCuucacugaa...	1	1	seq
....................cuuggUacugguagaauucacugaa...	6	1	seq
....................cuuggcacuggAagaauucacugaa...	5	1	seq
....................cuuggcacugguGgaauucacugaa...	5	1	seq
....................cuuggcacugguagaauucacCgaa...	2	1	seq
....................cAuggcacugguagaauucacugaa...	7	1	seq
....................cuuggcacugguagaauucacuUaa...	2	1	seq
....................cuCggcacugguagaauucacugaa...	5	1	seq
....................cuuggcacugguagaauucacAgaa...	4	1	seq
....................cuuggcGcugguagaauucacugaa...	5	1	seq
....................cuuggcacugguagUauucacugaa...	2	1	seq
....................cuuggcacugguagaauucacugaG...	7	1	seq
....................cuuggcacugguagaauucacugCa...	1	1	seq
....................cuuggcacugguagaauucacugUa...	4	1	seq
....................cuugCcacugguagaauucacugaa...	2	1	seq
....................cuuggcacugguagaUuucacugaa...	1	1	seq
....................cuuggcacuggCagaauucacugaa...	4	1	seq
....................cuuggcacugguagaauucacuNaa...	1	1	seq
....................cuuggcacugguagaauucacugaa...	11287	0	seq
....................cuuggcUcugguagaauucacugaa...	2	1	seq
....................cuuggcacugguagaaAucacugaa...	3	1	seq
....................Nuuggcacugguagaauucacugaa...	1	1	seq
....................cuuggcaAugguagaauucacugaa...	1	1	seq
....................cuuggcacugguagaauucaAugaa...	6	1	seq
....................cuuggcacugguagaauucacugaC...	3	1	seq
....................cuuggcacCgguagaauucacugaa...	3	1	seq
....................cuuggcacugguagaauuAacugaa...	4	1	seq
....................cuuggcacuCguagaauucacugaa...	1	1	seq
....................cuuggcacuAguagaauucacugaa...	11	1	seq
....................cuuggcacugguagaauucacugaU...	1048	1	seq
....................cuuggcacugguagaauuGacugaa...	4	1	seq
....................cuuggcacugguaCaauucacugaa...	1	1	seq
....................cuugAcacugguagaauucacugaa...	1	1	seq
....................cuuggcacugguUgaauucacugaa...	2	1	seq
....................cuuggcacugguagaauAcacugaa...	1	1	seq
....................cuuCgcacugguagaauucacugaa...	1	1	seq
....................cuuAgcacugguagaauucacugaa...	9	1	seq
....................cuuggAacugguagaauucacugaa...	5	1	seq
....................Auuggcacugguagaauucacugaa...	1	1	seq
....................cuuggcacugAuagaauucacugaa...	2	1	seq
....................cuuggcacAgguagaauucacugaa...	1	1	seq
....................cuuggcacugguagaauuUacugaa...	13	1	seq
....................cuuggcacugguagaaCucacugaa...	3	1	seq
....................cuuggcacugguagaaNucacugaa...	1	1	seq
....................cuuggcacugguagaauucacuAaa...	19	1	seq
....................cuuggcacugguagaauucacugGa...	2	1	seq
....................Uuuggcacugguagaauucacugaa...	8	1	seq
....................cuugUcacugguagaauucacugaa...	3	1	seq
....................cuuggGacugguagaauucacugaa...	1	1	seq
....................cuAggcacugguagaauucacugaa...	10	1	seq
....................cuuggcacugguagGauucacugaa...	2	1	seq
....................cuuggcacugguagaauuGcugaa...	4	1	seq
....................cuuggcacugguagaauucacuCaa...	1	1	seq
....................cuuggcacugguagaauucacugaau..	223	0	seq
....................cuuggcacugguagaauucacugaaA..	1006	1	seq

```
                    Mature                              Star
uaaaggcgcucccaacccuucuuggcacugguagaauucacugaauucuuuuaaguucagugaauuaccagucucaaaaauugugagugaaccauugcggcguacuuuacggac
....................cuuggcacugguagaauucacugaaC....................................................................   66     1   seq
....................cuuggcacugguagaauucacugaaG....................................................................   2      1   seq
....................cuuggcacugguagaauucacugaUu....................................................................   30     1   seq
....................cuuggcacugguagaauucacuUaau....................................................................   1      1   seq
....................cuuggcacugguagaauucacugaauu...................................................................   3      0   seq
....................cuuggcacugguagaauucacugaauA...................................................................   3      1   seq
....................cuuggcacugguagaauucacugaaAu...................................................................   2      1   seq
....................cuuggcacugguagaauucacugaUuu...................................................................   10     1   seq
....................cuuggcacugguagaauucacugaaGu...................................................................   4      1   seq
....................cuuggcacugguagaauucacugaauuUu.................................................................   1      1   seq
....................cuuggcacugguagaauucacugaauuUuu................................................................   3      1   seq
.....................uAggcacugguagaauuca..........................................................................   1      1   seq
.....................uuggcacugguagaauucac.........................................................................   10     0   seq
.....................uuggcacugguagaauucacu........................................................................   55     0   seq
.....................uuCgcacugguagaauucacu........................................................................   1      1   seq
.....................uuggcacugguagaauuUacu........................................................................   1      1   seq
.....................Cuggcacugguagaauucacug.......................................................................   1      1   seq
.....................uuggcacugguagaauucacug.......................................................................   94     0   seq
.....................uuggcacugguagaauucacuA.......................................................................   1      1   seq
.....................uuggcacugguagaauucacugG......................................................................   11     1   seq
.....................uuggcaUugguagaauucacuga......................................................................   1      1   seq
.....................uuggcacugUuagaauucacuga......................................................................   1      1   seq
.....................uuggcacugguagaauucacuga......................................................................   3800   0   seq
.....................uuggcacuAguagaauucacuga......................................................................   3      1   seq
.....................uuggcacugguagaauucaAuga......................................................................   1      1   seq
.....................uuggcacugguagaauucacugC......................................................................   1      1   seq
.....................uuggcacugguaAaauucacuga......................................................................   1      1   seq
.....................uuggcacugguGgaauucacuga......................................................................   2      1   seq
.....................uuggUacugguagaauucacuga......................................................................   2      1   seq
.....................uuggcacugguagaauUacuga......................................................................   3      1   seq
.....................uuggcacuggAagaauucacuga......................................................................   1      1   seq
.....................Auggcacugguagaauucacuga......................................................................   4      1   seq
.....................uuggcacugguagaauucacuAa......................................................................   2      1   seq
.....................uuggcacugguagaauuAacuga......................................................................   1      1   seq
.....................uuggcacugguagaauucacCga......................................................................   1      1   seq
.....................uuggcacugguagaauucCcuga......................................................................   1      1   seq
.....................uuAgcacugguagaauucacuga......................................................................   2      1   seq
.....................uugAcacugguagaauucacuga......................................................................   1      1   seq
.....................uuggcacugguagaauucacGga......................................................................   2      1   seq
.....................Cuggcacugguagaauucacuga......................................................................   12     1   seq
.....................uuggcUcugguagaauucacuga......................................................................   2      1   seq
.....................uuggcacugguagaauucacNga......................................................................   1      1   seq
.....................uuggcGcugguagaauucacuga......................................................................   3      1   seq
.....................uuggcacugguagaaAucacuga......................................................................   1      1   seq
.....................uNggcacugguagaauucacuga......................................................................   1      1   seq
.....................uuggcacugguagaauucUcuga......................................................................   1      1   seq
.....................uuggcacugguagUauucacuga......................................................................   1      1   seq
.....................uuggcaAugguagaauucacuga......................................................................   1      1   seq
.....................uuggcacuCguagaauucacuga......................................................................   1      1   seq
.....................uAggcacugguagaauucacuga......................................................................   1      1   seq
.....................uuggcacugguagaauucacAga......................................................................   1      1   seq
.....................Nuggcacugguagaauucacuga......................................................................   1      1   seq
.....................uuggcacugguagaauucacugU......................................................................   4      1   seq
.....................uuggcacugguagaauucacugaa.....................................................................   342    0   seq
.....................uuggcacugguagaauucacugaU.....................................................................   1      1   seq
.....................uuggcacugguagaauuAacugaa.....................................................................   1      1   seq
.....................uuggcacugguagaauucacuAaa.....................................................................   2      1   seq
.....................uuggcacugguagGauucacugaa.....................................................................   1      1   seq
.....................uuggcacugguagaGuucacugaa.....................................................................   1      1   seq
.....................uuggcacugguagaauucUcugaa.....................................................................   1      1   seq
.....................Auggcacugguagaauucacugaa.....................................................................   2      1   seq
.....................uuggcacugguagaauucacugaaA....................................................................   9      1   seq
.....................uuggcacugguagaauucacugaau....................................................................   1      0   seq
.....................uuggcacugguagaauucacugaaC....................................................................   2      1   seq
.....................uuggcacugguagaauucacugaauucu.................................................................   1      0   seq
......................uggcacugguagaauucacu........................................................................   5      0   seq
......................uggcacugguagaauucacug.......................................................................   10     0   seq
......................Cggcacugguagaauucacuga......................................................................   5      1   seq
......................uggcacugguagaauucacGga......................................................................   1      1   seq
......................uggcacugguagaauucacuga......................................................................   298    0   seq
```

```
                    Mature                                    Star
uaaaggcgcucccaacccuucuuggcacugguagaauucacugaauucuuuuaagguucagugaauuaccagucucaaaaauugugagugaaccauugcggcguacuuuacggac
......................uggcacugguagaauucacugG.......................................................................    1     1     seq
......................uggcacugguagaauucUcuga.......................................................................    1     1     seq
......................uggcacugguagaauucacugaa......................................................................    56    0     seq
......................Aggcacugguagaauucacugaa......................................................................    1     1     seq
......................uggcacugguagaauucacuAaa......................................................................    1     1     seq
......................uggcacugguagaauucacuAaau.....................................................................    1     1     seq
......................uggcacugguagaauucacugaaA.....................................................................    1     1     seq
......................uggcacugguagaauuAacugaau.....................................................................    1     1     seq
......................uggcaAugguagaauucacugaau.....................................................................    1     1     seq
......................uggcacugguagaauucacugaau.....................................................................    173   0     seq
......................Aggcacugguagaauucacugaau.....................................................................    1     1     seq
......................uggcacuAguagaauucacugaau.....................................................................    1     1     seq
......................uggcacugguagaauucacugaauu....................................................................    3     0     seq
......................uggcacugguagaauucacugaauuU...................................................................    1     1     seq
......................uggcacugguagaauucacugaauuc...................................................................    2     0     seq
.......................ggcacugguagaauucacuga.......................................................................    23    0     seq
.......................ggcacugguagaauuUacuga.......................................................................    1     1     seq
........................gcacugguagaauucacu.........................................................................    3     0     seq
........................gcacugguagaauucacug........................................................................    3     0     seq
........................gcacugguagaauucacAg........................................................................    1     1     seq
........................gcacugguagaauucacuga.......................................................................    69    0     seq
........................gcacugguagaauucacGga.......................................................................    5     1     seq
........................gcacuAguagaauucacuga.......................................................................    1     1     seq
........................Ucacugguagaauucacuga.......................................................................    1     1     seq
........................gcacugguagaauucacugaa......................................................................    2     0     seq
........................gcacugguagaauucacGgaa......................................................................    7     1     seq
........................gcacugguagaauucacugaauucu..................................................................    2     0     seq
.........................cacugguagaauucacug........................................................................    164   0     seq
.........................Uacugguagaauucacug........................................................................    1     1     seq
.........................cacugguagaauucacuU........................................................................    2     1     seq
.........................cacugguaUaauucacug........................................................................    1     1     seq
.........................cacuCguagaauucacuga.......................................................................    1     1     seq
.........................cGcugguagaauucacuga.......................................................................    1     1     seq
.........................cacugguagaauucacugG.......................................................................    5     1     seq
.........................cacugguagaGuucacuga.......................................................................    2     1     seq
.........................Uacugguagaauucacuga.......................................................................    2     1     seq
.........................cacugguagaauucacuCa.......................................................................    2     1     seq
.........................cacugguaCaauucacuga.......................................................................    1     1     seq
.........................cacugguaAaauucacuga.......................................................................    1     1     seq
.........................Aacugguagaauucacuga.......................................................................    1     1     seq
.........................cacugguagaauuUacuga.......................................................................    2     1     seq
.........................cacugguagaUuucacuga.......................................................................    1     1     seq
.........................Nacugguagaauucacuga.......................................................................    2     1     seq
.........................cacugguagaauucacuAa.......................................................................    3     1     seq
.........................cacugguagaauCcacuga.......................................................................    1     1     seq
.........................cacugguaUaauucacuga.......................................................................    1     1     seq
.........................cUcugguagaauucacuga.......................................................................    1     1     seq
.........................cacugguagaaAucacuga.......................................................................    2     1     seq
.........................cacugguagaauuAacuga.......................................................................    1     1     seq
.........................cacugguagaauucacGga.......................................................................    7     1     seq
.........................cNcugguagaauucacuga.......................................................................    1     1     seq
.........................cacugguagaauucacugU.......................................................................    14    1     seq
.........................cacugguagaauuGacuga.......................................................................    1     1     seq
.........................cacAgguagaauucacuga.......................................................................    4     1     seq
.........................cacugguagaauucacuga.......................................................................    2767  0     seq
.........................cacugguagaauucacugaa......................................................................    13    0     seq
.........................cacugguagaauucacGgaa......................................................................    1     1     seq
.........................cacugguagaauucacugaU......................................................................    1     1     seq
.........................cacugguaCaauucacugaa......................................................................    1     1     seq
.........................cacugguagaauucacugaaA.....................................................................    4     1     seq
..........................acugguagaauucacuga.......................................................................    653   0     seq
..........................acugguagaauucacGga.......................................................................    2     1     seq
..........................Gcugguagaauucacuga.......................................................................    7     1     seq
..........................acugguagaauucacuAa.......................................................................    1     1     seq
..........................acuggAagaauucacuga.......................................................................    1     1     seq
..........................acugguagaNuucacuga.......................................................................    1     1     seq
..........................Ccugguagaauucacuga.......................................................................    4     1     seq
..........................acugguagaauucacugU.......................................................................    2     1     seq
..........................acugguagaauuUacuga.......................................................................    2     1     seq
..........................acugguagaauuAacuga.......................................................................    1     1     seq
```

Mature   Star

```
uaaaggcgcucccaacccuucuuggcacugguagaauucacugaauucuuuuaaguucagugaauuaccagucucaaaaauugugagugaaccauugcggcguacuuuacggac
..........................acugguagaauucacugaU.....................................................................	2	1	seq
..........................acugguagaauucacugaa.....................................................................	1	0	seq
..........................acugguagaauucacGgaa.....................................................................	1	1	seq
..........................acugguagaauucacugaaA....................................................................	1	1	seq
...........................cugguagaauucacGgaa.....................................................................	4	1	seq
...........................cugguagaauucacugaa.....................................................................	34	0	seq
...........................cugguagaauucacugaU.....................................................................	13	1	seq
...........................cugguagaauucacugaG.....................................................................	1	1	seq
...........................cugguagaauucacugaau....................................................................	1	0	seq
...........................cugguagaauucacugaUu....................................................................	1	1	seq
...........................cugguagaauucacugaaA....................................................................	42	1	seq
..........................................................agugaauuaccagucucaaaa...................................	2	0	seq
..........................................................agugaauuaccagucucaaaaa..................................	3	0	seq
................................................................................uugugagugaaccauugcggc.............	1	0	seq
```
